# Supplementary material for: Integrated Analysis of the Lung Microbiome and Metabolome Reveals Associations Between Amino Acid Metabolism and Pulmonary Fibrosis in a Bleomycin-Induced Mouse Model
Source: Int J Mol Sci. 2026 Jun 30;27(13):5895. doi: 10.3390/ijms27135895 (PMC13362081; doi:10.3390/ijms27135895)
Supplement: Supplementary file 1 [file ijms-27-05895-s001.zip › result/4.MetDiffAnalysis/B_14d.vs.C_14d/ROC_all/Com_999_neg_ROC.pdf]

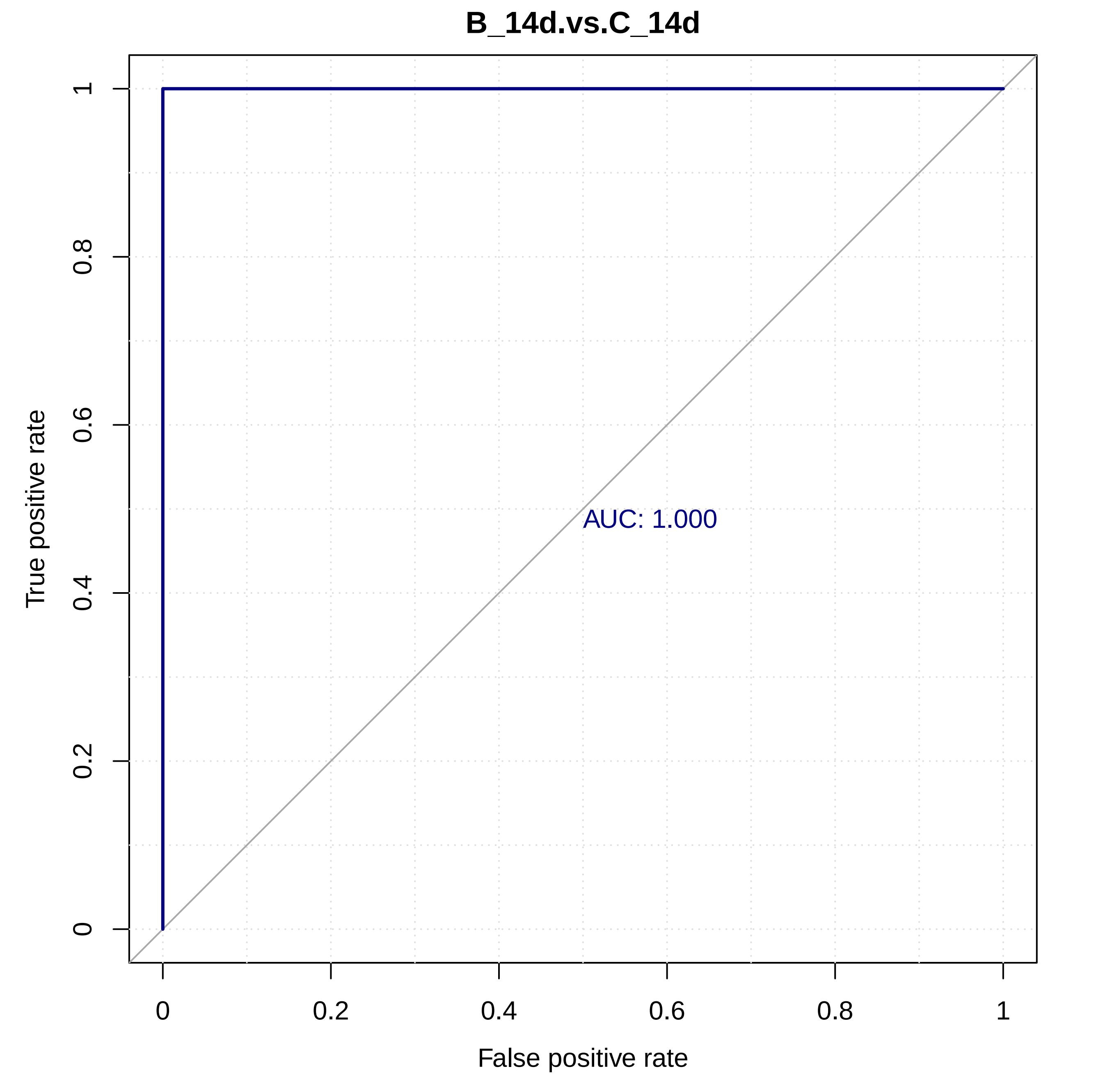
B_14d.vs.C_14d
AUC: 1.000
True positive rate
False positive rate
0
0.2
0.4
0.6
0.8
1
0
0.2
0.4
0.6
0.8
1
